# Multidrug-resistant toxigenic *Corynebacterium diphtheriae* sublineage 453 with two novel resistance genomic islands

**DOI:** 10.1099/mgen.0.000923

**Published:** 2023-01-27

**Authors:** Gabriele Arcari, Mélanie Hennart, Edgar Badell, Sylvain Brisse

**Affiliations:** ^1^​ Institut Pasteur, Université Paris Cité, Biodiversity and Epidemiology of Bacterial Pathogens, Paris, France; ^2^​ Department of Molecular Medicine, Sapienza Università di Roma, Rome, Italy; ^3^​ Collège doctoral, Sorbonne Université, F-75005 Paris, France; ^4^​ Institut Pasteur, National Reference Center for Corynebacteria of the Diphtheriae Complex, Paris, France

**Keywords:** *Corynebacterium diphtheriae*, diphtheria, toxigenic strains, multidrug-resistance, phylogeography, genomic epidemiology

## Abstract

Antimicrobial therapy is important for case management of diphtheria, but knowledge on the emergence of multidrug-resistance in *Corynebacterium diphtheriae* is scarce. We report on the genomic features of two multidrug-resistant toxigenic isolates sampled from wounds in France 3 years apart. Both isolates were resistant to spiramycin, clindamycin, tetracycline, kanamycin and trimethoprim-sulfamethoxazole. Genes *ermX, cmx, aph(3’)-Ib, aph(6)-Id, aph(3’)-Ic, aadA1, dfrA15, sul1, cmlA, cmlR* and *tet(33*) were clustered in two genomic islands, one consisting of two transposons and one integron, the other being flanked by two IS6100 insertion sequences. One isolate additionally presented mutations in *gyrA* and *rpoB* and was resistant to ciprofloxacin and rifampicin. Both isolates belonged to sublineage 453 (SL453), together with 25 isolates from 11 other countries (https://bigsdb.pasteur.fr/diphtheria/). SL453 is a cosmopolitan toxigenic sublineage of *C. diphtheriae,* a subset of which acquired multidrug resistance. Even though penicillin, amoxicillin and erythromycin, recommended as the first line in the treatment of diphtheria, remain active, surveillance of diphtheria should consider the risk of dissemination of multidrug-resistant strains and their genetic elements.

## Impact tatement

The genetic elements that carry antimicrobial resistance genes in *Corynebacterium diphtheriae* are not well documented and, until now, phylogenetic sublineages of toxigenic strains are considered as being geographically restricted.

In this study we uncovered the existence of a cosmopolitan toxigenic sublineage, a subset of which presented multiple antimicrobial resistance genes clustered in two genomic islands. This work contributes to a better understanding of the dynamics that underlie the spread of antimicrobial resistance in toxigenic *C. diphtheriae *.

## Data Summary

The complete genome sequence data for isolates FRC0137 and FRC0375 were deposited in the European Nucleotide Archive and are available at accessions GCA_902808445.2 and GCA_902808935.2, respectively.

## Introduction

Toxigenic *Corynebacterium diphtheriae* is responsible for diphtheria, a potentially severe infection of the upper respiratory tract that may include sore throat, neck swelling and distant toxinic signs. The pathognomonic symptom is the presence of a pseudomembrane, which adheres on the tonsils, oropharynx and pharynx. If untreated, the disease can potentially cause death from suffocation or due to distal toxic damage [1].

The incidence of diphtheria has dropped significantly following the Global immunization initiative [2]. Nevertheless, outbreaks of diphtheria can still occur when vaccination of populations is sub-optimal, as observed in the ex-Soviet Union countries in the 1990s [3] and more recently in the Rohingya [4, 5] and Yemeni populations [6].

Diphtheria therapy may include the timely administration of diphtheria anti-toxin (DAT), but this product is in short supply [7]. Antimicrobial treatment is central in clinical case management and for contacts' prophylaxis, and typically comprises oral or parenteral penicillin, amoxicillin or erythromycin. Even though antimicrobial resistance is more frequent in some other *Corynebacterium* species [1], *C. diphtheriae* isolates with reduced susceptibility towards penicillin, cefotaxime and erythromycin as well as tetracycline, sulphonamide or other agents have been reported [2, 8–10].

Yet, knowledge on the epidemiological links and dissemination of resistant *C. diphtheriae* is scarce. *C. diphtheriae* is phylogenetically structured into multiple sublineages, and most of them currently appear geographically restricted [6, 11, 12]. However, rather than true endemism, this may reflect both under-sampling and the lack of systematic genomic sequencing in microbiology laboratories. There are also large gaps in the understanding of the genomic mechanisms underlying antimicrobial resistance (AMR) in this species, and the physical organization of AMR genes within *C. diphtheriae* genomes is largely unknown except for early work on a few strains, e.g. [13]. Some recent studies have reported the presence of transposons [14] or of a plasmid [9] carrying a *pbp2m* transfer unit, sometimes associated with the *ermX* methyltransferase gene. Other members of the *Corynebacterium* genus harbour transposons, insertion sequences and plasmids that carry AMR genes [15–17].

In a previous study of the population structure and antimicrobial-resistance phenotypes of a large collection of *C. diphtheriae* [9], several multidrug-resistant (MDR) isolates were observed; MDR was defined as resistance to antimicrobial agents belonging to three or more categories – excluding fosfomycin to which all *Corynebacterium* are resistant [18]. Here, we investigate further two MDR isolates, FRC0137 and FRC0375. After long-read sequencing and genome-sequence completion, a focus was put on the genomic context of their resistance elements. Furthermore, we aimed to place the two isolates in their evolutionary context by analysing publicly available genomes that belong to the same *C. diphtheriae* sublineage (SL), SL453.

## Methods

Isolates FRC0137 and FRC0375 were sampled in 2012 and 2015, respectively. The identification took place at the National Reference Center for Corynebacteria of the *diphtheriae* complex (NRC-CCD) as previously described [9] using PCR assays and MALDI-TOF mass spectrometry (Bruker). The presence of the diphtheria toxin *tox* gene was determined by PCR [9] and confirmed by blastN of the DIP_ RS12515 reference *tox* gene on the genomic sequences and a more recent real-time PCR assay [19]. The production of the toxin was assessed using the modified Elek test [20].

In addition, the isolates were characterized biochemically for pyrazinamidase, urease, nitrate reductase, for utilization of maltose and for glycogen fermentation. This allowed determining their biovar, based on the combination of nitrate reductase (positive in Mitis and Gravis, negative in Belfanti) and glycogen fermentation (positive in biovar Gravis only).

Phenotypic susceptibility to antimicrobial agents was tested by disc diffusion (BioRad, Marnes-la-Coquette, France) for the antibiotics listed in Table 1. When possible, the results were interpreted according to CA-SFM/EUCAST V.1.0 (January 2019) [21]. In other cases, the interpretative criteria published in Table III of the CA-SFM 2013 recommendations [22] were applied.

**Table 1.** Antimicrobial resistance phenotypes and genotypes of isolates FRC0137 and FRC0375

Antibiotic class	Antimicrobial agent	Diameter (mm) of FRC0137	Diameter (mm) of FRC0375	Breakpoint (EUCAST 2019) [21]	Resistance element (FRC0137)	Resistance element (FRC0375)
**β lactams**	**Penicillin (1 UI**)	**17**	**20**	29	None detected	None detected
	**Amoxicillin**	46	35	23	None detected	None detected
	**Oxacillin**	30	26	20	None detected	None detected
	**Cefotaxime**	37	28	26	None detected	None detected
	**Imipenem**	57	44	24	None detected	None detected
**Macrolides**	**Erythromycin**	32	31	22	*ermX*	*ermX*
	**Clarithromycin**	42	33	22	*ermX*	*ermX*
	**Azithromycin**	**21**	27	22	*ermX*	*ermX*
	**Spiramycin**	**22**	**11**	24	*ermX*	*ermX*
**Lincosamides**	**Clindamycin**	**14**	**6**	20	*ermX*	*ermX*
**Streptogramin**	**Pristinamycin**	42	40	22	None detected	None detected
**Aminoglycosides**	**Kanamycin**	**6**	**6**	17	*aph(3’)-Ib, aph(6)-Id, aadA1*	*aph(3’)-Ib, aph(6)-Id, aadA1*
	**Gentamicin**	31	27	23	None detected	None detected
**Rifamycin**	**Rifampicin**	**6**	35	30	S444F in rpoB *	None detected
**Tetracycline**	**Tetracycline**	**15**	**18**	24	*tet(33*)	*tet(33*)
**Quinolone**	**Ciprofloxacin**	**7**	33	25	S90F and D94Y in gyrA †	None detected
**Folate pathway inhibitors**	**Sulfonamide**	**6**	**6**	17	*sul1*	*sul1*
	**Trimethoprim**	**6**	**6**	16	*dfrA15*	*dfrA15*
	**Trimethoprim-Sulfamethoxazole**	**6**	**6**	19	*sul1, dfrA15*	*sul1, dfrA15*
**Fosfomycin**	**Fosfomycin**	**6**	**6**	14	Intrinsic resistance	Intrinsic resistance
**Glycopeptide**	**Vancomycin**	27	19	17	None detected	None detected
**Oxazolidinone**	**Linezolid**	34	35	25	None detected	None detected

*Same of *Corynebacterium urealyticum* in [38].

†Same position, different substitutions compared with *C. urealyticum* in [38].

The strains were previously [9] sequenced with a NextSeq-500 instrument (Illumina, San Diego, CA, USA) at a minimum of 50×coverage depth; trimming was performed using AlienTrimmer v0.4.0 [23], redundant or over-represented reads were reduced using the khmer software v1.3 [24], sequencing errors were corrected using Musket v1.1 [25] and a *de novo* assembly was performed for each strain using SPAdes v3.12.0 [26]. The quality of the assembly generated by SPAdes was assessed using BUSCO [27]. Here, the two isolates were also subjected to Oxford Nanopore Technologies (ONT) sequencing, for which genomic DNA was extracted using a phenol-chloroform approach. Libraries were prepared using a 1D ligation sequencing kit (SQK-LSK-108) without fragmentation and sequenced using a MinION FLO-MIN-106 flow cell.

ONT and Illumina short reads were combined to generate a hybrid assembly using Unicycler v0.4.4 [28] (normal assembly mode, default parameters).

Genome annotation was performed using rast [29]. The genomes were screened for resistance and plasmid replicon genes using the ResFinder 4.1 [30] and PlasmidFinder 2.1 [31] online tools at the https://cge.cbs.dtu.dk/services/ website. Synteny between the resistance regions of the FRC0137 isolate and homologous regions from other genomes was analysed by mapping blastN alignments with the Circos visualization tool [32]. A neighbor-joining tree was generated based on the concatenated nucleotide sequences of the 1305 genes of the cgMLST scheme [11] of SL453 isolates using the https://bigsdb.pasteur.fr/diphtheria/ platform, and the tree was visualized using iTOL [33].

## Results

### Characteristics of the isolates and description of resistance genomic islands RGI-I and RGI-II

Isolates FRC0137 and FRC0375 were sampled from wound infections of two patients living in a single mainland France city. Strain FRC0137 was isolated in 2012 from a foot abscess in a patient with a recent hospitalization in South Asia. The wound was first treated by administration of amoxicillin-clavulanic acid for 7 days, and later on with vancomycin and ceftriaxone, which was followed by the resolution of the wound infection. Isolate FRC0375 was isolated in 2015 from an ankle ulceration in a patient who had also travelled to the same South Asian country but no earlier than 10 years before the isolation of the strain; in this case the infection was treated with an undefined macrolide and no further details on the outcome were available.

Phenotypic characterization showed that both isolates belonged to biovar Mitis (nitrate positive, glycogen negative) and produced the diphtheria toxin, as determined by Elek’s test. Antimicrobial susceptibility testing showed that both isolates were resistant to clindamycin, spiramycin, kanamycin, tetracycline and trimethoprim-sulfamethoxazole; furthermore, FRC0137 was resistant to azithromycin, rifampicin and ciprofloxacin (Table 1). Both isolates were susceptible to erythromycin and clarithromycin even though they harboured the *ermX* gene (see below). They also appeared penicillin-resistant, as do most *C. diphtheriae* using the currently proposed breakpoint [9].

High-quality genome assemblies of FRC0137 and FRC0375 were obtained, with a size of 2 549 354 bp and of 2 613 747 bp, and G+C % content of 53.63 and 53.62 %, respectively. The FRC0137 genome was assembled into two contigs whereas the genome sequence of FRC0375 was fully circularized. No plasmids were identified. Sequence analysis confirmed the biochemical characterization of toxigenicity and biovar: the two isolates carried an identical, non-disrupted copy of the *tox* gene (allele 2 in the BIGSdb database, https://bigsdb.pasteur.fr/diphtheria/) and were negative for the Gravis biovar-associated *spuA* gene region [9, 34].

**Fig. 1.**
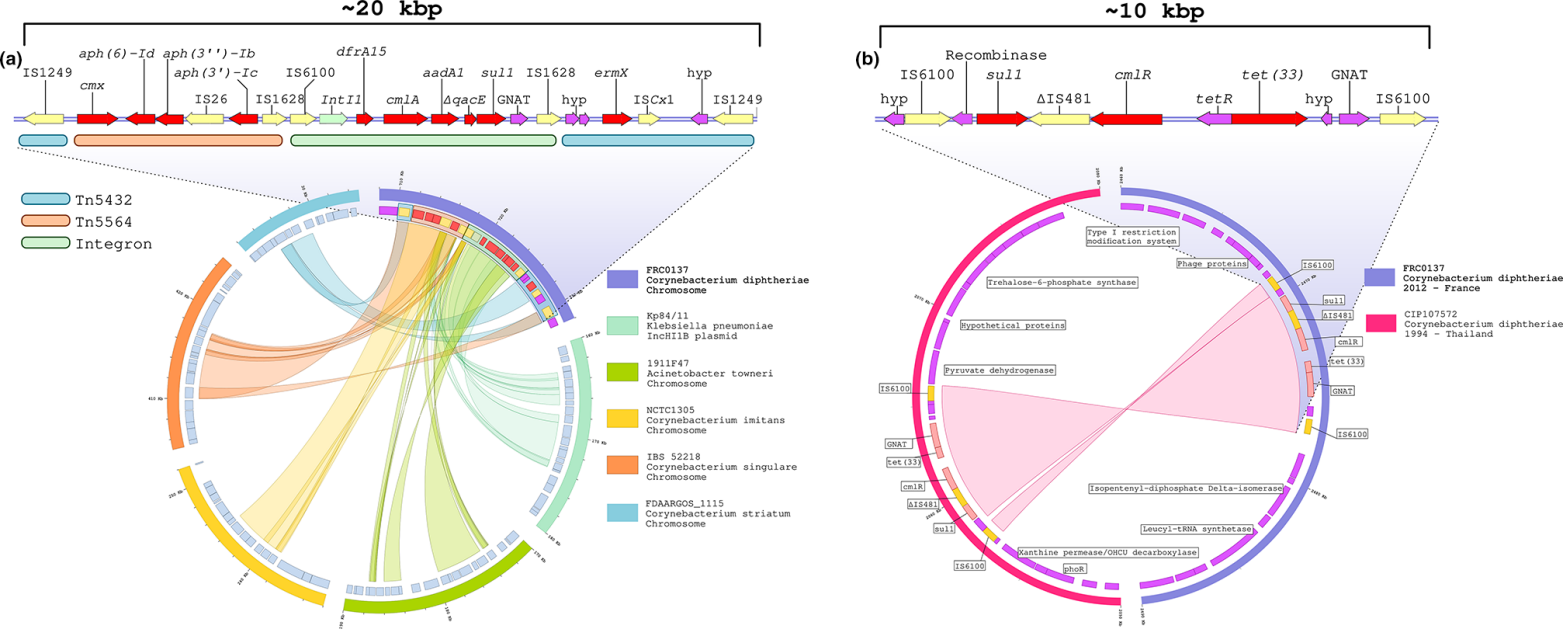
Schematic representation of the *Cd*-RGI-I and *Cd*-RGI-II resistance islands (a). The upper part represents a map of the approximately 20 kbp, which make up *Cd*-RGI-I of the FRC0137 isolate. Arrows indicate the genes and are colour coded as follows: red for antimicrobial resistance genes, yellow for ISs, green for integrase genes and purple for miscellaneous genes. Coloured horizontal bars below the genes indicate the several parts that compose the resistance genomic island: blue, the fragmented Tn5432; orange, Tn5564; and green, the integron. In the lower part, the synteny with several other isolates retrieved from the GenBank database is shown. Genes are colour-coded as in (a). Specifically, the Tn5432 alignment with a *Corynebacterium striatum* isolate is depicted in blue; the Tn5564 alignment with a *Corynebacterium singulare* is depicted in orange and the one with *Corynebacterium imitans* in yellow. The integron alignments' regions are drawn in green with the IncHI1B plasmid of *Klebsiella pneumoniae* and in aquamarine with the chromosome of *Acinetobacter towneri *. (b) The upper part represents a map of the approximately 10 kbp, which makes up the *Cd*-RGI-II of isolate FRC0137. Arrows indicate the genes and are colour coded as in (a). In the lower part, is shown a synteny plot with *C. diphtheriae* CIP107572 isolated from Thailand in 1994. Beyond the loss of a small portion of the recombinase gene in FRC0137, these two islands have 100 % sequence identity. Note that the genomic context in which the islands are located is different, indicating the horizontal transfer of the island.

The genomes of both isolates carried 12 antimicrobial resistance genes, which we found were grouped on two antimicrobial resistance islands. The first one, herein named *Cd*-RGI-I, had a length of 20 769 bp in strain FRC0137 (Fig. 1a) and of 19 328 bp in strain FRC0375. The difference in size is due to the loss of the remnants of an IS*Cx*1 in the latter isolate. The second resistance island was named *Cd*-RGI-II and was of size 8 570 bp in both strains (Fig. 1b). *Cd*-RGI-I contained nine antimicrobial resistance genes, plus a GCN5-related N-acetyltransferase family (GNAT) gene and a truncated version of the *qacE* gene. This resistance island may be divided into three distinct elements (Fig. 1a). The first element corresponds to the transposon Tn5432, which is composed by two IS1249 genes belonging to the IS256 family, and the macrolide resistance gene *ermX* as well as IS*Cx*1. The latter, absent in the FRC0375 strain, may be considered as a genomic scar, since it is prematurely interrupted by a stop codon [17]. Transposon Tn5432 has already been described in other *Corynebacterium* species with some variation [15, 17].

The second element composing this resistance island is transposon Tn5564, which is bounded by an IS5564. It comprises the *cmx* gene coding for a chloramphenicol efflux pump, and an IS6 family member IS1628 gene. Tn5564 also includes the *aph(3’)-Ib* and *aph(6)-Id* (formerly known as *strA* and *strB*) streptomycin resistance genes and the *aph(3’)-Ic* aminoglycoside phosphotransferase, which was potentially imported with Tn5564 [15, 17] .

The third element of *Cd*-RGI-I is composed by an integron flanked by two insertion sequences (IS6100 and IS1628), and comprises four gene cassettes corresponding to AMR genes (*dfrA15, cmlA, aadA1* and *sul1*), *ΔqacE* and a GNAT gene. Hence, this element may underlie the resistance to trimethoprim, sulfamethoxazole, chloramphenicol and aminoglycosides (*aad1*: streptomycin and spectinomycin). It was not possible to trace back this integron to any previously described *Corynebacterium* or Actinobacteria (GenBank database, last accessed date 7 December 2021). Rather, it may come from Gram-negative species, given that the best sequence matches (maximum 82 % coverage and 99 % identity) were observed with Gamma-proteobacteria such as *Escherichia coli* (LT985260.1), *Klebsiella pneumoniae* (CP030270.1, KX029332.1) and *Acinetobacter species* (CP046045.1, CP046596.1). In *Cd*-RGI-I, transposon Tn5432 might have been the first element to integrate into the chromosome of an ancestral *C. diphtheriae,* and may then have served as a ‘hotspot’ for the integration of the two other elements present in the region, as already proposed [35].

The second resistance island (*Cd*-RGI-II) is flanked by two IS6100 insertion sequences and may thus correspond to a transposon; it was identical (100 % identity, 100 % length coverage) in both isolates. It carries a recombinase gene followed by the *sul1* gene in a tail-to-tail configuration, by a deleted IS481, and by two other AMR genes: *cmlR* and *tet(33*), potentially contributing to the observed resistance to chloramphenicol and tetracycline (Fig. 1b). We found that this island is present with the same composition but in a different genomic context in a phylogenetically distant (1050 different cgMLST alleles out of 1305 gene loci) *tox*-positive *C. diphtheriae* isolate CIP107572, which belongs to MLST sequence type ST258 and was isolated in Thailand in 1994 [9, 36, 37].

FRC0137 differed from FRC0375 by being resistant to two additional antimicrobial agents: rifampicin and ciprofloxacin. Consistently, in FRC0137 mutations in the genes encoding for the β-subunit of the RNA polymerase (*rpoB*) and for the gyrase subunit A (*gyrA*) were observed. FRC0137 displayed a deduced S444F alteration of the RpoB protein, previously described in a rifampicin-resistant *Corynebacterium urealyticum* [38]. However, this specific mutation did not affect the *rpoB* MLST allele, as it lied outside of the *rpoB* region used in MLST; three additional, synonymous changes were observed within the *rpoB* MLST template, leading to allele rpoB-13 (ST136) compared to FRC0375 *rpoB* sequence (allele 2, ST389).

The amino acid alterations S90F and D94Y deduced from the *gyrA* gene sequence probably underlie the observed ciprofloxacin resistance in FRC0137. *C. urealyticum* isolates displaying S90V and D94Y amino-acid substitutions were also resistant to quinolones [38], and both phenylalanine and valine have a hydrophobic side chain known to favour the disruption of the water–metal ion bridge [39].

### Evolution within sublineage SL453

**Fig. 2.**
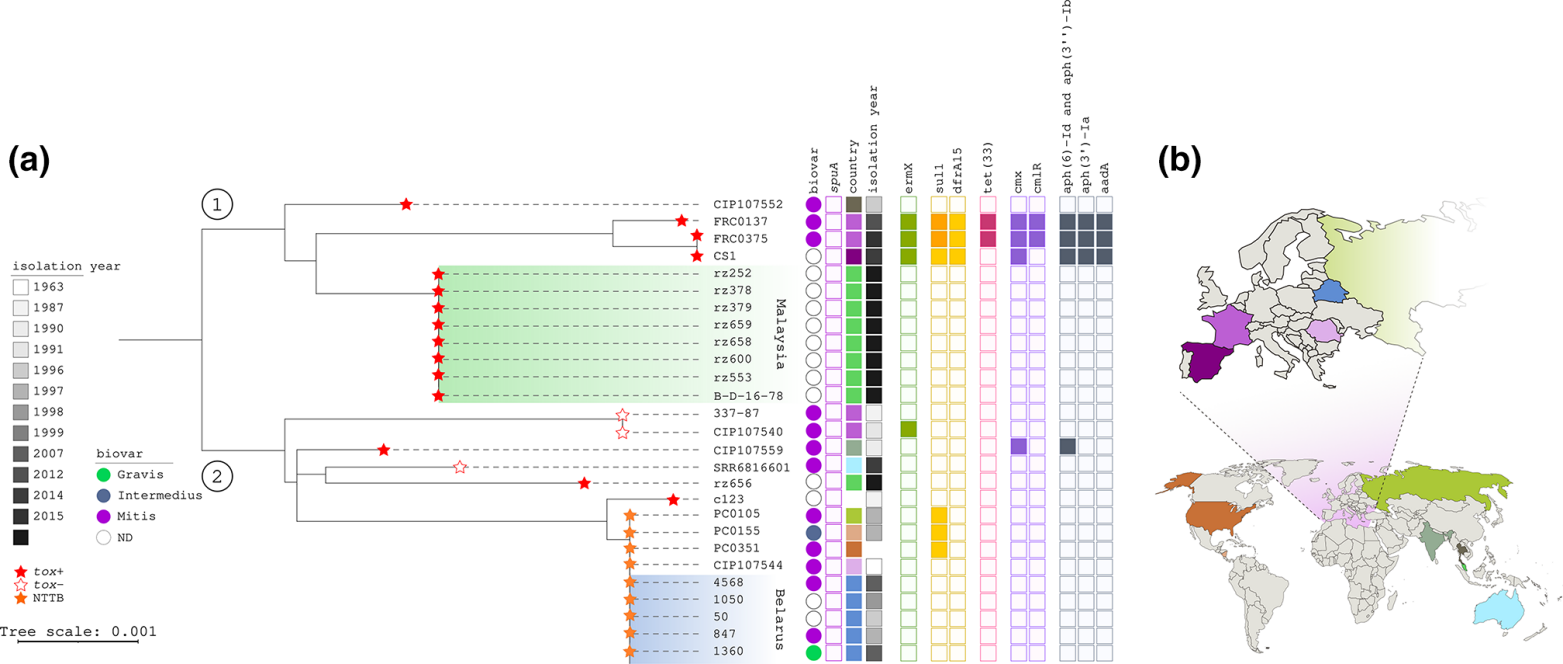
Phylogeny of *Corynebacterium diphtheriae* sublineage 453, and distribution of isolates features (a). Midpoint-rooted neighbour-joining tree based on the concatenated nucleotide sequences of the 1 305 genes of the cgMLST scheme of *C. diphtheriae *. Nodes are colour-coded as follows: empty stars indicate the absence of the *tox* gene, orange stars indicate non-toxigenic toxin bearing (NTTB) isolates, and red stars indicate *tox* positive, toxigenic isolates. Other data are colour-coded according to the keys or, for resistance genes: empty squares indicate the absence of the gene, filled squares indicate the presence of the gene, and a darker colour indicates isolates harbouring two copies of the specific gene (*sul1* in this case). The scale indicates the number of nucleotide substitutions per site. (b) World map download from https://simplemaps.com/resources/svg-world displaying the country of origin of SL453 isolates. The image was edited using the OpenSource InkScape software.

To understand the evolutionary origins of multidrug-resistance in these isolates, we performed a comparative genomic analysis. According to cgMLST [11], they differed by 131 loci, and thus belong to the same sublineage, SL453 [11]. We found that 25 publicly available genomes also belonged to SL453. These were sampled from 1963 to 2016, nine of them from Malaysia [40], two from France [9, 41], five from Belarus [42], two from Spain [43] and one each from Australia, Guatemala, India, Romania, Russia, Thailand and the USA (Fig. 2b). One isolate had an unknown provenance.

We defined two branches within SL453 based on its phylogenetic structure (Fig. 2). Most SL453 isolates (24/27) carried the *tox* gene even though nine isolates of branch 2, associated with the 1990s outbreak in Belarus (Fig. 2), were defined as non-toxigenic toxin-bearing (NTTB) isolates as deduced from *tox* gene disruption and as previously reported for some of them [42]. Only three isolates of branch 2 harboured an intact copy of the *tox* gene, and three did not carry it. Branch 1 isolates were all hypothesized as being toxigenic, based on their complete *tox* gene, and had diverse geographical and temporal origins: the older isolate was from Thailand, 1996, while eight isolates were from a 2016 outbreak in Malaysia. The three other branch 1 isolates were from Europe, including FRC0137, FRC0375 and isolate CS1 from Spain. CS1 was isolated from a young patient having a travel history in Afghanistan [43] and belonged to the same outbreak cluster (i.e. less than 25 allelic mismatches [11] as FRC0375. Thus, the latter isolate from France is genetically closer from isolate CS1 from Spain, than from FRC0137. Notably, isolate CS1 lacks the *Cd*-RGI-II resistance island, suggesting an initial acquisition of this island in the ancestor of the three isolates, followed by its loss in the isolate from Spain.

## Discussion

Toxigenic *C. diphtheriae* outbreaks have been reported in recent years [5, 6, 44–46], and occurred in countries with disrupted healthcare services, which do not achieve high vaccination coverage [1]. Antimicrobial treatment is an important addition to diphtheria antitoxin treatment, which is often unavailable and only targets the toxin effects. Therefore, antimicrobial resistance in toxigenic strains is a matter of public health concern.

Here we analysed two MDR toxigenic isolates and discovered two genomic islands that together carry 12 resistance genes and which were previously unreported in *C. diphtheriae,* to our knowledge. Even though most of the resistance genes they carried, except for *ermX*, may confer resistance to antimicrobial agents that are not recommended as he first line in the treatment of *C. diphtheriae* infection or colonization, the resistance profile of these two isolates has public health relevance. First, these genomic regions may in the future aggregate more AMR genes and disseminate them to other sublineages of *C. diphtheriae* or other *Corynebacterium* species. Second, these resistance genes may serve as markers for the yet untraced spread of MDR toxigenic *C. diphtheriae *. The fact that the resistance islands carry genes that confer resistance to agents that are not used against diphtheria, highlights the risk of long-term maintenance of resistance genes, either in the absence of antimicrobial selective pressure, or due to bystander selection of resistance in *C. diphtheriae* through exposure to antibiotics used to treat other infections [47].

The international spread of SL453 isolates and the evolutionary pattern of the RGI-I and RGI-II provide clues as to the scenario of their dissemination. The two isolates from France, and the one from Spain, were linked to South Asia. The acquisition of the resistance islands Cd-RGI-I and Cd-RGI-II might therefore have occurred in this world region. In fact, many MDR isolates previously described in *C. diphtheriae* were linked to this region [10, 12]. Acquisition of the RGIs in SL453 must have occurred after the evolutionary split from the lineage leading to the Malaysian outbreak strain, which does not carry them. We hypothesize that the common ancestor of the three RGI-I carrying isolates had acquired the two RGIs, with subsequent loss of RGI-II in the branch leading to isolate CS1. The three isolates have unexpected genetic relationships, with a close genetic identity of isolate CS1 with isolate FRC0137, which was isolated from a patient in the same French city as FRC0375. The lack of fit observed in this case between epidemiology and genomics illustrates the value of genomic epidemiology and underlines the need to improve the rate of *C. diphtheriae* infection reporting and microbiological characterization to better trace the links between sporadic infections.

Another isolate belonging to SL453, of ST136 (the same ST as FRC0137), was described in Canada in 2011, from a patient who returned from India with a toe infection [48]; and the antimicrobial susceptibility pattern of this isolate was similar to that of FRC0375. No other ST136 isolate was reported from India or elsewhere [12].

The FRC0137 isolate, isolated 3 years before the FRC0375 one, displayed a mutated *gyrA*, indicating that evolution towards quinolone resistance happened in the branch leading to FRC0137, potentially under quinolone selective pressure in Asia [49, 50]. Microevolution of *Corynebacterium* quinolone resistance in defined epidemiological contexts has been observed previously [6], underlying the high risk of emergence of resistance to this antimicrobial class during treatment in *C. diphtheriae* and other *Corynebacterium* species.

A previous study has shown that most sublineages harbouring the *tox* gene have a limited geographical distribution [9]. Our study, instead, uncovers the existence of a cosmopolitan toxigenic MDR clone. We concur with previous statements [11, 12] that *C. diphtheriae* is currently sampled at a largely insufficient rate to decipher its geographical spread with confidence, and it is to be expected that, as *C. diphtheriae* genomic epidemiology develops, more sublineages with broad spatial distribution will be described. Genomic surveillance of *C. diphtheriae* should be strengthened globally, in order to monitor the spread of sublineages of particular concern such as the MDR toxigenic SL453.
